# Nurses' Experiences of Conflict Management at a Teaching Hospital in Namibia: A Qualitative Study

**DOI:** 10.1155/2023/6663194

**Published:** 2023-12-12

**Authors:** Takaedza Munangatire, Nestor Tomas, Maria M. Alweendo

**Affiliations:** University of Namibia, P.O. Box 88, Rundu, Namibia

## Abstract

The purpose of this study was to explore nurses' experiences of conflict management at a central hospital in Namibia. Conflict in nursing practice is inevitable and experienced regularly. Understanding how nurses experience conflict is at the centre of the successful handling of conflict, which may enhance teamwork and quality of patient care. A phenomenological transcendental (descriptive) phenomenology design was used in this study. Fifteen nurses were purposively selected at the teaching hospital and interviewed to generate data, which were analysed thematically between June and November 2021. Five themes were generated from a rigorous analysis process: understanding of conflict and conflict management; development of conflict; conflict management approaches; consequences of conflict; and in-service training. Conflict situations that nurses face during their practice may remain unresolved or inappropriately resolved if they lack the necessary education and skills in conflict management. Nursing education and continuous education on conflict management should thus be provided to all nurses to empower them to manage conflict situations, which could improve their nursing practice and quality of patient care. There is a need to strengthen conflict management education in both undergraduate and continuous education programmes for nurses.

## 1. Introduction

Conflict is defined as the actual or perceived opposition of needs, values, and/or interests between two or more people, which is caused by individual or organisational factors, resulting in unwanted stress, tension, or negative feelings between the disputants [1]. In conflict, there is a continuous process of disagreement and antagonism within or between social groups, including professionals [2]. A conflict can be a result of actual threats, or when people think that there is a threat to their interests [3]. When there is a conflict, there is an obvious need to manage it, especially in the context of nursing care which relies on effective team work for quality patient care.

Extant research on conflict management has largely focused on nurses in leadership and managerial positions, yet practicing nurses are typically at the centre of most conflicts in the workplace. According to Jerng et al. [4], nurses report conflictual encounters more than any other professional, making management of nurses a challenging task. By its nature, nursing practice predisposes nurses to conflict situations that can emanate from practice differences, cultural divergences, organisational problems, distinct competencies, and a shortage of resources, among others [5–8]. In Namibia, nurses make up approximately 80% of the healthcare workforce, due to a lack of other healthcare professionals. This leaves nurses at a high risk of job stress [9]. With this burden of work, coupled with the factors mentioned above, nurses in Namibia commonly experience burnout [10]. While burnout has been reported as a potential outcome of conflict, it is also possible that nurses who are burnt out are avoiding managing conflict [11]. The situation described above suggests that conflict is ever present, unavoidable, and sometimes necessary in the practice of nursing [12, 13]. There is a particular need to focus on conflicts among staff members who are on a similar level, as this type of conflict is likely to affect the quality of patient care more than others [14, 15]. Managing conflict is thus necessary to ensure the promotion of teamwork in patient care, as well as to enhance nurses' leadership skills [16, 17].

The experience of conflict can be positive or negative leading to a variety of outcomes [8, 13, 17]. There is evidence that some junior nurses lack conflict management skills, and when conflict is unresolved, it creates a toxic working environment with negative consequences for the quality of patient care [18–21]. On the other hand, when conflict is managed successfully, it generates a conducive working environment with good teamwork among nurses and other healthcare professionals [22].

Nurses apply different styles when managing conflict, which can result in both positive and negative outcomes [23, 24]. Some researchers suggest that avoidance is the most commonly used conflict management style [6, 18], yet others argue that avoidance is considered the least used style [8, 14]. According to Saridi et al. [12], nurses typically opt to compromise in conflict situations, while other studies reveal that although nurses do collaborate in conflict solving, there is also a tendency to accommodate [25]. There is thus no conclusive evidence regarding the conflict management styles used by nurses and their managers, making it an important area for further research. It is also critical to assess the human experience of the conflict management process, the findings of which could form the basis of better and more constructive conflict management [26].

According to Moeta and Du Rand [24], most nurse managers are competent in managing conflict, but nonmanagerial nurses lack this capacity; hence, they require support to improve in this regard. While all nurses globally are educated on conflict management, it is not clear to what extent it prepares junior nurses and nonmanagerial nurses [14]. In Namibia, conflict management is part of the nursing curriculum; however, as a soft skill, there is no evidence of how the development of this skill is fostered in Namibia beyond theoretical knowledge. In terms of research, conflict and conflict management are areas that have not been sufficiently explored; hence, there is a need for further studies in the context of nursing. In their study, Labrague et al. [8] asserted that practicing nurses need education and team-building activities to improve their conflict management skills, while a study by Sexton and Orchard [22] showed that training on conflict management predicts healthcare professionals' perceived ability to resolve conflicts. Similarly, Ylitörmänen et al. [27] explained that there is a need to develop nurses' conflict management skills if they are to resolve conflicts constructively.

In order to help nurses develop conflict management skills, it is necessary to explore how they experience the conflict process. People's experiences are important because they shape their perceptions and their future behaviours. By using experience as the basis for educating nurses about conflict, there is likely to be alignment in what they need and education they get. The purpose of this study was thus to explore nurses' experiences of conflict management at a central hospital in Namibia.

## 2. Methods

### 2.1. Approach and Design

A qualitative phenomenological transcendental (descriptive) phenomenology design was used in this study. This research approach focuses on describing a phenomenon as it is lived [28, 29]. The emphasis is on describing the way in which people comprehend a phenomenon, what they experience, how they experience it, and the meaning that the phenomenon gives to their experience [30].

### 2.2. Sampling Strategy

The study was carried out at a university teaching hospital in Windhoek, Namibia, which is also a referral hospital. The hospital has a bed capacity of 830 beds. It acts as a general referral hospital for the country and the main teaching hospital admitting pediatric and adult patients with medical, surgical, and maternal-related conditions. The hospital has a capacity to employ at least 1000 nurses of different categories and specialisations. The researchers assumed that all of the hospital's nurses had experienced conditions that exposed them to conflict at some point in their work environment; hence, all of them were considered eligible for the study. The researchers then purposively selected participants to generate a sample of maximum variation in terms of years of experience, gender, qualification, and department. This type of sampling was suitable because it helped the researchers to identify common and variable features of the phenomenon of conflict, as experienced by a variety of nurses in various departmental contexts [31]. The sample size was determined by data sufficiency, which was reached with 15 participants of mixed gender, age, experience, and department. Three researchers were involved in an iterative data analysis process, which allowed for an adequate interpretation of the data [32]. In reality, the size of a sample is not as important as the quality of the data collected and their ability to answer the research questions and ultimately generate meaningful interpretive themes [33].

### 2.3. Ethical Issues

Ethical clearance was obtained from the University of Namibia School of the Nursing Ethics Committee and the Ministry of Health Research Ethics Committee (So NEC 07/2021 and 17/3/3 MAA), and permission to carry out the study was received from the teaching hospital. Since experiences of conflict may include sensitive issues that could personal, the researchers gave participants an option to be referred for psychosocial support if they wished so.

### 2.4. Data Collection Methods

The author (MA) who was working at the hospital approached the nurses in charge of the different units to seek access to the practicing nurses. Information about the study was shared, and those who expressed an interest in participating went through the informed consent process and physically signed an informed consent form. A total of 25 nurses agreed to participate in the study, but ultimately, only 15 were interviewed because data saturation was attained. In-depth, face-to-face individual interviews were used to collect data between June and November 2021. All the interviews were conducted in English as the participants were comfortable with the language. Each interview was tape-recorded using a voice recorder. Private rooms at the hospital were used as the venue for the interviews as they provided privacy and were quiet.

The first interview was a pilot interview, which was analysed to test the suitability of the interview guide to generate relevant data as well as to create the initial coding framework. No significant changes needed to be made to the questions in the interview guide; thus, the pilot interview was retained as part of the main data. Suggestions were made to add more probing questions to capture additional aspects of the nurses' experience of conflict management.

Subsequently, one interview after another was conducted, with each interview being transcribed and analysed by the three researchers (MA, TM, and NT) before the next interview was conducted. This was done to ensure that rich data were collected and to detect initial evidence of data saturation, which was reached at the 12^th^ interview. Three additional interviews were conducted for confirmation of data saturation. Data sufficiency was evidenced by the participants' interviews not providing any new information that was significantly different to the data provided in previous interviews. Furthermore, the concurrent data collection and analysis enabled the researchers to detect code sufficiency, as the interviews were no longer generating any new codes. Last, the researchers' interpretation was also used to determine sufficiency, where all three researchers independently coded and interpreted the data by looking at the same interviews at different times and blindly coding the data. When there were no substantial new codes or interpretations, saturation was considered to be reached.

### 2.5. Data Collection Instruments and Technologies

A semistructured interview guide was used to guide the interviews (supplementary file 1), which was developed by the researchers based on the research objective. There were two sections: section A included questions on the participants' demographics, while section B included open-ended questions to allow the participants to express themselves as much as they could. The main question was as follows: “What were your experiences in resolving conflict situations you encountered in your workplace?”

While this was the key question, follow-up questions were asked to explore the whole structure of the experience in detail. These included the following: “What actions did you take? Explain how you reacted? What emotions were you going through? How did these emotions influence your actions or reactions in the short and long term?”

The development of these questions was guided by the literature, which was clearly lacking information regarding the experience of conflict and its management among nonmanagerial nurses.

### 2.6. Data Processing

Soon after each interview, the data were transferred to the password-protected computer of one of the researchers, who then transcribed the data verbatim and shared the transcript with the other researchers. These emails were permanently deleted, and the documents were saved onto password-protected personal computers. The interviewees were named using codes that were not identifiable.

### 2.7. Data Analysis

The data were analysed using content analysis. The steps of this analysis were decontextualisation, recontextualisation, categorisation, and compilation [34]. Following the verbatim transcriptions, the researchers independently read the transcripts and listened to the interviews while making notes of their general impressions. During the decontextualisation process, the researchers read the data in order to extract meaning units based on the participants' descriptions of their experiences (actions, reactions, feelings and thoughts, and changes in these over time). Based on these meaning units, the researchers generated codes that were a mixture of both the participants' exact words (manifest analysis) and the researchers' interpretations (latent analysis) [34]. In the second stage, recontextualisation, the codes were compared with the original interview transcripts to ensure that they were supported by the data. The researchers discussed their codes, which included providing explanations for how each reached their decisions. During the discussions, some new codes emerged, which were added to the existing codes.

After agreeing that the researchers had generated sufficient codes, more latent analysis was applied with a focus on uncovering the underlying meaning of the data related to the nurses' experiences of conflict (categorisation process). This process resulted in the grouping of codes based on similarities or some linkages in terms of explaining the nurses' experiences of conflict management. Further critical analysis resulted in the formation of categories and themes, which were named and explained with supporting quotations. The researchers deliberately promoted divergence throughout the data analysis process to ensure that there was a move towards analysis saturation. While the authors do not claim that meaning unit, code, category, and theme saturation were reached, some form of data sufficiency was attained, and realistic conclusions were reached based on the data (see supplementary file 2 for sample data analysis).

### 2.8. Trustworthiness

The study applied Lincoln and Guba's [35] criteria for ensuring trustworthiness, i.e., credibility, confirmability, and dependability. Credibility was ensured through researcher triangulation during data analysis and member checking, while confirmability was attained by using the participants' own words to support the created themes. To ensure dependability, the Standards for Reporting Qualitative Research (SRQR) tool was used, which created an auditable trail of the methods and processes applied in this study [36]. In terms of reflexivity, one researcher was an employee at the hospital where the study was conducted, while the other two were employed at the teaching institution. The researcher (MA) who was working with the participants collected the data; hence, it is possible that some relationships may have influenced the data collected despite the researcher's attempt to avoid this. The other two researchers may have influenced the data and analysis, and therefore the findings, during the analysis process and ultimately the writing of the findings. The authors therefore declare that the data and findings of this study are not only based on the participants' views but also on the authors' philosophical assumptions and interpretations of the data. It is impossible to entirely exclude the researchers in qualitative research, as they drive it from conceptualisation to conclusion [37, 38]. Two of the authors (TM and NT) are experienced researchers in qualitative research, with previous training in interviewing and data analysis. MA was trained in data collection before starting the data collection process, which was supervised and monitored from interview to interview.

## 3. Results and Discussion

### 3.1. Participants' Characteristics

Of the 15 participants who were interviewed, eight were female and seven were male. Of the 15 participants, 12 fell into the age group of 21 to 30; the remaining three were aged between 31 and 40. Twelve of the participants had degrees in nursing science, while three had diplomas in nursing. Each participant had work experience of at least two years.

### 3.2. Nurses' Experiences of Conflict Management

**Figure 1:**
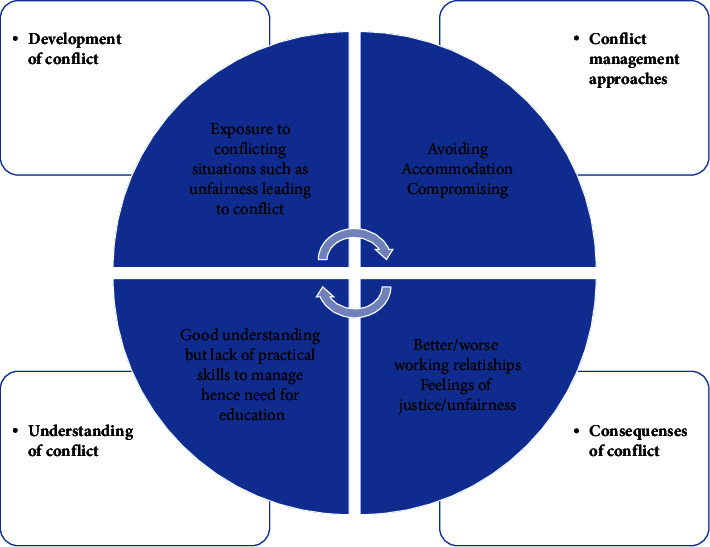
Nurses' experiences of conflict management.

The findings of this study are diagrammatically presented in Figure 1. These findings are then discussed in detail below the diagram.

#### 3.2.1. Theme 1: Understanding of Conflict and Conflict Management

This theme focused on the participants' understanding of conflict, i.e., what it is and what it means to them. While understanding conflict is not an experience, ascertaining this was important because the way someone experiences something can be influenced by their understanding of it. Therefore, in interpreting the participants' experiences of conflict, their understanding of the subject could help provide some explanation. The participants understood conflict as a misunderstanding or disagreement between two or more people who fail to agree or reach an understanding on a certain issue, who may need the assistance of a third party to resolve the matter. In these disagreements, arguments may occur which can be verbal or physical, resulting in a loss of peace and harmony within the work environment.

*“Conflict is a misunderstanding between two or more people, or it can be a misunderstanding between a group of people whereby they can't reach an agreement on something, or maybe they don't get along due to a certain problem they come across.” (P3)*

*“Well in my own understanding, conflict simply means the disagreement between, for instance, two or more parties with different opinions, needs or interests. Conflict can actually result into major arguments, so to say physical abuse or definitely loss of peace and harmony within the work environment; that's how I understand it.” (P9)*

Regarding conflict management, some participants understood it as a peace-making process that can be achieved through negotiation in a fair manner. On the other hand, some considered avoiding conflict as a way of conflict management.

*“Okay, well, I was actually prepared for any kind of reaction when I approached her so I knew she might react in a good or bad way, but then I felt I was at a position where I need to avoid unnecessary confrontations” (P4)*

This theme confirmed that the participants were knowledgeable about what conflict is. The subsequent themes described their experiences of conflict management.

#### 3.2.2. Theme 2: Development of Conflict

The collaborative nature of nursing care predisposes nurses to the development of conflict between themselves, their managers, other healthcare professionals, patients, and relatives of patients. The nurses felt that conflict is inevitable in a nursing working environment but argued that it is not always a bad thing in the end. They stated that how one interprets the actions or motives behind another person's actions can result in conflict.

*“Conflict is unavoidable because there are situations [in which] people need to correct each other or act for the good. However not everyone will interpret the actions in a good way, hence leading into some conflict.” (P15)*

*“Conflict or disagreement will never make you feel good, but as long as you know you are doing it for a good cause….” (P3)*

*“It was very sad and emotional for me because you are being blamed for things that are totally not your fault and something that is out of your control.” (P2)*

Conflict can arise when nurses are correcting each other, as some people are unhappy when they are corrected or if someone uses a negative approach to correct them. Such disagreements, when not solved, can build momentum towards conflict. Sometimes it is not that nurses do not accept being corrected, but there is poor communication, which may lead to conflict when people either explicitly display their emotions or do so through subtle actions.

*“Okay, let me see if I can mention one that I had; it's a disagreement about drug recording. My colleague was failing to record or she was not recording the drugs that she uses on the patient and when I informed her about it, or to improve on her record keeping, she disagreed with me that she is doing her job and she is recording her things.” (P9)*

*“Poor communication skills of nurses lead to interpersonal conflicts, and in most cases, we are afraid to express our opinions out of fear of being condemned, so we engage in contrary actions.” (P1)*

Feelings of being treated unfairly and a lack of transparency can make those who are excluded from certain activities or decision-making feel bitter and be silently conflictual. As reported in the excerpt below, the aggrieved party started to act in negative ways to attract attention and display their displeasure. This is an avoidance of the situation, but it attracts attention, which can result in confrontation and hence expose the conflict.

*“Actually, what I have figured out… most causes of conflict can be maybe when people are going through something and they are not speaking out, probably, favouritism at the workplace, poor communication or selective listening, or… maybe poor or bad attitudes.” (P12)*

*“So, one of the nurses I found already working there started constantly coming late to work and then when I approached her, she reacted defensively and claimed that I was made the acting matron out of favouritism.” (P3)*

*“I had a disagreement with a co-worker over delegation; this specific co-worker felt like she was overworked or she was given too many tasks that she could not handle.” (P12)*

#### 3.2.3. Theme 3: Conflict Management Approaches

The nurses' experiences of conflict management revealed that a range of approaches can be applied to deal with conflict, including avoiding it and confrontation. When avoiding conflict, some nurses do not see the issue as not being important, but they would rather avoid talking about it, so they stop talking to the person they consider has wronged them, and the other person does not do anything in return.

*“I remember I had a conflict with one of my colleagues and it's kind of brought hatred, whereby the person stopped talking to me just because I made the off duty the way he didn't want the off duty to be made, regardless of whatever reason that I gave.” (P15)*

One nurse's experience demonstrated an accommodating way of dealing with conflict. By listening to why one nurse was always late, it made her understand the situation better but did not necessarily solve the problem.

*“In my conflict situation I asked her how I could assist her improving maybe her time managing, so she actually calmed down and told me that she doesn't have a nanny at home so she was actually getting late with regards to kids' preparation for school and so forth.” (P4)*

There seems to be an understanding among some nurses that conflict can be solved through compromises, where both parties have to take some responsibility for the problem.

*“You have to compromise; you have to listen to other parties and you both parties have to compromise; to come to one conscience whereby you agree.” (P13)*

*“Yes, well, the conflict was resolved as we all came to an agreement or a conclusion; we just came to an adjustment that we were both wrong since we were both supposed to be responsible in this case.” (10)*

Other conflict management circumstances had the nurses failing to resolve a conflict as each side wanted to be a winner and disregarded the interests of the other side. There was a denial of responsibility regarding the cause of the conflict, with the blame being pushed towards the other side. In cases where third parties were involved, one nurse viewed them as biased. Such conflict management processes created bad situations and left some nurses disappointed and feeling a sense of injustice.

*“There was one colleague of mine that was not really happy with the off duty, she felt like some people are favoured and she is overworked, so it was not easy; it was really bad and we had a fight; that is one of the conflicts I was involved in.” (P1)*

*“In most of the conflicts that I was involved in, I wasn't at fault; I am just trying to do what is right to be done, so I don't really feel bad about it because you know at the end of the day it is just work-related things–I do my job at work.” (P10)*

In some situations, the parties involved felt they were respected and given an opportunity to talk to each other and explain their sides of the story regarding the conflicting matters. The participants described their feelings as good and positive and said that they were satisfied with the conflict resolution process.

*“She let everyone of us to talk; she was really not taking sides, she listened attentively and she was trying to rule out or to figure out where the problem really came from. The other thing she was also not interrupting between our conversations, she made sure that everyone had a say and everyone had to explain what really happened.” (P1)*

*“When we started the conflict management process I felt good; I was feeling positive because of the fact that we were all willing to listen to each other, we were all willing to find a solution that can work for both of us.” (P12)*

However, in some cases, the conflicting parties felt the process was unfair because the negotiator took sides. While in the long run the conflict was resolved, having a negative experience during the process could discourage participants from engaging other parties to solve conflicts.

*“It was a bit disappointing; somehow, we also thought that the management were siding with our superiors so we felt like we were not being heard. It wasn't an easy experience throughout the process… but luckily in the end we reached consensus and we agreed upon one thing. It felt better and we are good now.” (P13)*

#### 3.2.4. Theme 4: Consequences of Conflict

The nurses had experienced both negative and positive outcomes with conflict management. In cases where the resolution process went smoothly, the parties felt good and motivated to engage in dealing with the conflict. However, in some circumstances, the conflict management process resulted in hatred, leaving the parties in a worse working relationship than before.

*“I actually felt bad because I personally don't like to be in conflict; I don't like to be involved in conflict because it's also emotionally draining but it is part of life. We encounter conflict almost on daily basis.” (P15)*

*“You find that you get in a conflict despite you being right, the person will hate you or you find that somebody will love you for correcting them or for bringing up something, but in my experience it's really a harsh one.” (P13)*

The experiences of the nurses suggested that conflict is unavoidable. When conflict occurs and is resolved appropriately, however, it can culminate in new and better ways of understanding work and improved working relationships. The nurses' experiences suggested that when a common understanding exists, some causes of conflict such as supportive supervision can lead to improved quality of work.

*“My experience is to say, I can say conflict is not always something to fear… it is not really always a bad thing because when resolved properly it can lead to better ideas; you know better understanding or even better working relationships.” (P5)*

*“She improved, I showed changes, she kept up her recording and if you remind her she does not get angry like she used to do before, meaning she understood why we have to do our record keeping and record our drugs.” (P3)*

Some nurses experienced conflict as an opportunity to get to know each other in terms of how one thinks or feels about different situations; it is only through understanding others that good relationships can develop. Although this should happen in better ways than conflict, sometimes it takes conflict for it to happen.

*“It is very good to have disagreement in order for you to understand the ground of another person and the views of another person, because this gives you also the opportunity to open up and talk to the person openly about how you feel about a situation that you don't like.” (P13)*

#### 3.2.5. Theme 5: In-Service Training

Conflict experienced by nurses is rooted in the nursing care activities and how these are handled among the nurses. Participants in this study suggested that there is a need for training on aspects of managing emotions such as anger, as well as education on carrying out nursing activities such as delegation. Conflict management should be part of the nursing education, they argued, not only at school but in the workplace. The approach to conflict management education should include role plays showing the necessary steps needed to resolve conflict.

*“So, for instance, let me say nurses need probably in-service training or workshops on anger management and doing tasks such as delegating effectively.” (P6)*

*“Definitely my point. I think if it starts from school, like if there could be content about conflict management at school, and we continue with it at the workplace through in-service training.” (P7)*

*“…this training if it's possible maybe to be done as short role plays just on conflict or misunderstandings between two people, whereby steps are given or certain procedures are being given to sisters on how to come about and how to solve the problem.” (P10)*

## 4. Discussion

### 4.1. Understanding of Conflict and Conflict Management

In general, the interviews showed that the nurses had a good understanding of conflict and conflict management. However, in this qualitative study, the purpose was not to objectively quantify their knowledge but rather to use their understanding to help explain how they experience conflict and conflict management.

### 4.2. Development of Conflict

The way the nurses described their experiences of conflict management in this study suggests that differences in personalities and how people interpret the actions or motives of others are at the centre of conflict. It is this nature of conflict that supports the assertion that conflict is always present and inevitable in the practice of nursing [12, 13]. In addition, situations that likely evoke people's emotions or expose their weaknesses are experienced as sources of conflict. This is in line with the findings of Gi and Ki [5], who noted that differences in competency between nurses become sources of conflict. While fairness is a subjective construct, the way nurses feel about being treated fairly was reported as one of the causes of conflict in this study. For this reason, conflict management education should include understanding nurses' emotions on the matter of fairness in nursing activities and opportunities at work. These findings support the assertion by Freedman [39] that fairness, given its complicated and subjective nature, is a source of conflict. This study showed that tasks such as duty rosters, which directly affect nurses, can cause conflict if nurses are not involved in their development. According to Lee et al. [40], the high involvement of staff in work practices helps to improve relationships and reduce conflict.

### 4.3. Conflict Management Approaches

The literature highlights how nurses apply different conflict management styles and classifies them into different categories. The findings of this study confirm that nurses do use different conflict management styles, as presented in previous studies [8, 14]. Some of the nurses' experiences of conflict management suggest a continuum, where nurses start dealing with conflict by avoiding it before managing it through collaboration. The findings of this study have added additional details regarding nurses' use of conflict management styles; for example, avoidance was reported as the most common conflict management style [6, 18] as the nurses want to keep the peace, so they tolerate what they are not happy about. According to Leodoro et al. (2018), accommodation is another style used by some nurses.

This study demonstrated that empathy is the reason behind some nurses' use of accommodation in conflict management. One nurse described putting themselves in another person's shoes and ending up tolerating what would otherwise not be acceptable to them. These styles seem to keep the peace without solving the conflict; however, the more differences persist, the more likely there is to be confrontation. This could result in either direct competition or accommodation. Such outcomes in conflict management are similar to those reported by O'Toole et al. [41], who argued that the ways in which conflict is handled can result in conflict being managed, resolved, or transformed.

### 4.4. Consequences of Conflict

Nurses' experiences of conflict management in this study showed that conflict is not always solved and that the process could actually result in more conflicts. This is as per some previous studies, which have demonstrated that not all conflict is successfully resolved [23, 24]. Based on the findings of this study, it can be said that nurses' management of conflict is not based on which style to use, but it is entrenched in their experiences of encountering and dealing with conflict. By understanding the nurses' experience of the conflict process, the focus on conflict management styles should shift to understanding the underlying processes that inform the way nurses deal with conflict.

The findings in this study show than even when nurse managers act as negotiators in conflict management, the outcome is not always positive. While the need for training, as suggested by Labrague et al. [8], cannot be ignored, there are additional underlying reasons for poor conflict management than a lack of education. A root cause analysis of the contributing factors to the development and mismanagement of conflict should be conducted as a holistic approach to conflict management education, thereby controlling the outcome of the conflict management process.

### 4.5. In-Service Training

The findings of this study suggest that nurses need formal education on conflict and conflict management. Using strategies such as role playing can improve their knowledge and skills. This is as per Arveklev et al. [42, 43], who noted that through drama, nurses' skills in conflict management could be improved. The need for education on conflict management, especially among nonmanagerial nurses, was also emphasised by Moeta and Du Rand [24], while Wigert et al. [15] suggested that conflict management should be used as a learning strategy in nursing. While education alone has been shown to help improve nurses' perceptions of their ability to solve conflict [22, 27], it is not adequate if nurses continue to fail to practice fairness and competence in performing some of duties. In addition, the nurses' experiences revealed that conflict and its management trigger emotions that are not controlled by education but emotional intelligence. A study by Hadman et al. [44] proposed that educating nursing managers about emotional intelligence could improve their conflict management skills. While emotional intelligence will not automatically improve conflict management [45], it is a tool that can be used in the constructive management of conflict. Ultimately, nurses need more education on conflict and managing conflict situations [42].

## 5. Conclusion

Conflict situations that nurses face during their practice may remain unresolved or inappropriately resolved if they lack the necessary education and skills to manage them. Nursing education and ongoing education on conflict management should thus be provided to all nurses to empower them to manage conflict situations..

## 6. Implications for Nursing Education and Practice

Nurses are prone to conflict situations due to the nature of their job, among other factors; thus, there is a need for educational interventions—both in undergraduate curricula and in continuous professional education—to foster development of their conflict management skills.

## Data Availability

The data that support the findings of this study are available at reasonable request from the authors and with permission from the ethical bodies.

## Supplementary Materials

Supplementary Materials (1) The interview guide is attached as the supplementary file. (2) The data analysis sample shows quotations and codes generated and how codes were grouped into categories and ultimately a theme.

## References

[1] Almost J., Wolff A. C., Stewart‐Pyne A., McCormick L. G., Strachan D., D’Souza C. (2016). Managing and mitigating conflict in healthcare teams: an integrative review. *Journal of Advanced Nursing*.

[2] Rahim M. A. (2011). *Managing Conflict in Organizations*.

[3] Johansen M. L. (2012). Keeping the peace: conflict management strategies for nurse managers. *Nursing Management*.

[4] Jerng J. S., Huang S. F., Liang H. W. (2017). Workplace interpersonal conflicts among the healthcare workers: retrospective exploration from the institutional incident reporting system of a university-affiliated medical center. *PLoS One*.

[5] Gi H., Ki Y. (2018). Hospital nurses’ experiences of conflict and conflict resolution. *Korean Journal of Occupational Health Nursing*.

[6] Kaitelidou D., Kontogianni A., Galanis P. (2012). Conflict management and job satisfaction in paediatric hospitals in Greece. *Journal of Nursing Management*.

[7] Sinskey J. L., Chang J. M., Shibata G. S., Infosino A. J., Rouine-Rapp K. (2019). Applying conflict management strategies to the pediatric operating room. *Anesthesia and Analgesia*.

[8] Labrague L. J., Al Hamdan Z., McEnroe‐Petitte D. M. (2018). An integrative review on conflict management styles among nursing professionals: implications for nursing management. *Journal of Nursing Management*.

[9] Pieters W. R., Matheus L. (2020). Improving general health and reducing burnout of nurses in Namibia. *SA Journal of Human Resource Management*.

[10] Ashipala D. O., Nghole T. M. (2022). Factors contributing to burnout among nurses at a district hospital in Namibia: a qualitative perspective of nurses. *Journal of Nursing Management*.

[11] Dall’Ora C., Ball J., Reinius M., Griffiths P. (2020). Burnout in nursing: a theoretical review. *Human Resources for Health*.

[12] Saridi M., Panagiotidou A., Toska A., Panagiotidou M., Sarafis P. (2021). Workplace interpersonal conflicts among healthcare professionals: a survey on conflict solution approach at a General Hospital. *International Journal of Healthcare Management*.

[13] Broukhim M., Yuen F., McDermott H. (2019). Interprofessional conflict and conflict management in an educational setting. *Medical Teacher*.

[14] Bajwa N. M., Bochatay N., Muller-Juge V. (2020). Intra versus interprofessional conflicts: implications for conflict management training. *Journal of Interprofessional Care*.

[15] Wigert H., Berg L., Arveklev S. H., Morrison-Helme M., Lepp M. (2021). Managing conflict situations nursing students encounter during their clinical practice, narrated and performed through Forum Play. *Nurse Education in Practice*.

[16] Pines E. W., Rauschhuber M. L., Cook J. L. (2014). Enhancing resilience, empowerment and conflict management among baccalaureate students. *Nurse Educator*.

[17] Özkan Tuncay F., Yaşar Ö., Sevimligül G. (2018). Conflict management styles of nurse managers working in inpatient institutions: the case of Turkey. *Journal of Nursing Management*.

[18] Lahana E., Tsaras K., Kalaitzidou A., Galanis P., Kaitelidou D., Sarafis P. (2019). Conflicts management in public sector nursing. *International Journal of Healthcare Management*.

[19] Alshehry A. S. (2022). Nurse–patient/relatives conflict and patient safety competence among nurses. *Inquiry: The Journal of Health Care Organization, Provision, and Financing*.

[20] Kim S., Buttrick E., Bohannon I., Fehr R., Frans E., Shannon S. E. (2016). Conflict narratives from the health care frontline: a conceptual model. *Conflict Resolution Quarterly*.

[21] Kim S., Bochatay N., Relyea-Chew A. (2017). Individual, interpersonal, and organisational factors of healthcare conflict: a scoping review. *Journal of Interprofessional Care*.

[22] Sexton M., Orchard C. (2016). Understanding healthcare professionals’ self-efficacy to resolve interprofessional conflict. *Journal of Interprofessional Care*.

[23] Kim W., Nicotera A. M., McNulty J. (2015). Nurses’ perceptions of conflict as constructive or destructive. *Journal of Advanced Nursing*.

[24] Moeta M. E., Du Rand S. M. (2019). Using scenarios to explore conflict management practices of nurse unit managers in public hospitals. *Curationis*.

[25] Labrague L. J., McEnroe-Petitte D. M., Papathanasiou I. V. (2018). Stress and coping strategies among nursing students: an international study. *Journal of Mental Health*.

[26] Koesnell A., Bester P., Niesing C. (2019). Conflict pressure cooker: nurse managers’ conflict management experiences in a diverse South African workplace. *Health SA Gesondheid*.

[27] Ylitörmänen T., Kvist T., Turunen H. (2015). A web-based survey of Finnish nurses’ perceptions of conflict management in nurse-nurse collaboration. *International Journal of Caring Sciences*.

[28] Neubauer B. E., Witkop C. T., Varpio L. (2019). How phenomenology can help us learn from the experiences of others. *Perspectives on Medical Education*.

[29] Wertz F. J. (2016). Outline of the relationship among transcendental phenomenology, phenomenological psychology, and the sciences of persons. *Schutzian Research*.

[30] Teherani A., Martimianakis T., Stenfors-Hayes T., Wadhwa A., Varpio L. (2015). Choosing a qualitative research approach. *Journal of Graduate Medical Education*.

[31] Suri H. (2011). Purposeful sampling in qualitative research synthesis. *Qualitative Research Journal*.

[32] Vasileiou K., Barnett J., Thorpe S., Young T. (2018). Characterising and justifying sample size sufficiency in interview-based studies: systematic analysis of qualitative health research over a 15-year period. *Bone Marrow Concentrate Medical Research Methodology*.

[33] Johnson J. L., Adkins D., Chauvin S. (2020). A review of the quality indicators of rigor in qualitative research. *American Journal of Pharmaceutical Education*.

[34] Bengtsson M. (2016). How to plan and perform a qualitative study using content analysis. *NursingPlus Open*.

[35] Lincoln Y., Guba E. G. (1985). *Naturalistic Inquiry*.

[36] De Jong Y., van der Willik E. M., Milders J. (2021). A meta-review demonstrates improved reporting quality of qualitative reviews following the publication of COREQ-and ENTREQ-checklists, regardless of modest uptake. *Bone Marrow Concentrate Medical Research Methodology*.

[37] Olmos-Vega F. M., Stalmeijer R. E., Varpio L., Kahlke R. (2023). A practical guide to reflexivity in qualitative research: AMEE Guide No. 149. *Medical Teacher*.

[38] Varpio L., Ajjawi R., Monrouxe L. V., O’Brien B. C., Rees C. E. (2017). Shedding the cobra effect: problematising thematic emergence, triangulation, saturation and member checking. *Medical Education*.

[39] Freedman B. D. (2019). Risk factors and causes of interpersonal conflict in nursing workplaces: understandings from neuroscience. *Collegian Journal*.

[40] Lee Y., Lee H. (2015). Role conflict and conflict management styles of hospital nurses. *Korean Journal of Stress Research*.

[41] O’Toole J., Bagshaw D., Burton B. (2019). *Researching Conflict, Drama and Learning: The International DRACON Project*.

[42] Arveklev S. H., Berg L., Wigert H., Morrison-Helme M., Lepp M. (2018). Learning about conflict and conflict management through drama in nursing education. *Journal of Nursing Education*.

[43] Arveklev S. H., Berg L., Wigert H., Morrison-Helme M., Lepp M. (2018). Learning about conflict and conflict management through drama in nursing education. *Journal of Nursing Education*.

[44] Al‐Hamdan Z., Adnan Al‐Ta’amneh I., Rayan A., Bawadi H. (2019). The impact of emotional intelligence on conflict management styles used by Jordanian nurse managers. *Journal of Nursing Management*.

[45] Ranjbar M., Bahariniya S. (2021). The relationship between emotional intelligence and conflict management in healthcare systems: a case study in Iran. *British Journal of Healthcare Management*.

