# Supplementary material for: Nurses' Experiences of Conflict Management at a Teaching Hospital in Namibia: A Qualitative Study
Source: J Nurs Manag. 2023 Dec 12;2023:6663194. doi: 10.1155/2023/6663194 (PMC11918614; doi:10.1155/2023/6663194)
Supplement: Supplementary Materials — (1) The interview guide is attached as the supplementary file. (2) The data analysis sample shows quotations and codes generated and how codes were grouped into categories and ultimately a theme. [file 6663194.f1.zip › CONFLICT STUDY-INTERVIEW GUIDE.docx]

# ANNEXURE A: INTERVIEW GUIDE

**TITLE: EXPERIENCES OF REGISTERED NURSES REGERDING CONFLICT MANAGEMENT**

**Introduction**

- Introduce self to participants by giving full name and surname
- Summarise the purpose of study being conducted and that participant is voluntary
- Confirm willingness to continue participating and assure participants on right to withdraw, privacy, confidentiality as espoused in the information sheet and consent form.
- Indicate estimated duration of the interview

**Section A: Demographic data**

- Ask about: Age, gender, years of experience, highest qualification.

**Section B: Main questions**

1. What is your understanding of conflict?

2. Describe a conflict situation you have been involved in during your practice as a nurse at this hospital?

3. What were your experiences in resolving these conflict situations?

- *what actions did you take; explain how you reacted; what emotions were you going through; how did these emotions influence your actions or reactions in the short and long term”.*

4. What do you think can be done to help registered nurses manage conflict in a better way?

***Possible follow up/probing questions***

- Explain in more detail
- Explain what you mean
- Tell me more
- What did you feel or think about it?

Thank you so much, we have come to the end of our interview.
