# Supplementary material for: Nurses' Experiences of Conflict Management at a Teaching Hospital in Namibia: A Qualitative Study
Source: J Nurs Manag. 2023 Dec 12;2023:6663194. doi: 10.1155/2023/6663194 (PMC11918614; doi:10.1155/2023/6663194)
Supplement: Supplementary Materials — (1) The interview guide is attached as the supplementary file. (2) The data analysis sample shows quotations and codes generated and how codes were grouped into categories and ultimately a theme. [file 6663194.f1.zip › Data analysis sample (1).docx]

Below is a sample on how the data was analyzed , showing the quotations, codes generated, how codes were grouped into categories and ultimately a theme.

| **Quotations from interview transcripts** | **Generated codes** | **Categories** | **Theme** |
| --- | --- | --- | --- |
| *P2: For me a conflict is an argument or misunderstanding between two individuals which could possible last longer than expected, sometimes might need other party or individuals to come in to help resolve it.*  *P3: Conflict is a disagreement between two people, two colleagues, two partners, is called conflict while the process of trying to resolve this difference is conflict management which serves to bring back good relations*  *P4: Well in my own understanding, conflict simply means the disagreement between for instance two or more parties with different opinions, needs or interest conflict can actually result into major arguments so to say physical abuse or definitely loss of peace and harmony within the work environment, that’s how I understand it. Also , any measures taken to bring the people to an agreement and forgive each and be in good books again is part of conflict management*  *P5: Personally I will define conflict as a disagreement between two people or two groups of people at a certain place. This conflict can only end if the people confront each other and openly share their concerns and find a common ground. In some cases, like in my case we had to use our manager who acted as a go between to resolve our differences.*  *P15: I do understand conflict as a disagreement between two or more than two parties and conflict can be good and also can be bad. Good in that , it helps people get to know each other better and if the conflict is over their relationship may become better but it is also bad because it can cause enmity and people will not work together well in the ward.*  *P14: Well conflict to my understanding is a misunderstanding if I should put it in better words, is when two people or more have misunderstanding or fight for lack of better word or don’t agree yes is a disagreement, don’t agree on something and it results in them maybe having a verbal argument or a physical fight so basically is a disagreement that turn into can lead to fight.* | Misunderstanding  Lack of agreement  Long term  Verbal  Physical | **Nature of conflict** | Understanding of conflict and conflict management |
|  | Differences in interests  Underlying issues  Differences in opinions | **Cause of conflict** |  |
|  | Individuals  Groups  Involved parties  Two parties  Third party | **Parties** |  |
|  | Problem solving  Consensus  Resolving | **Management** |  |
|  | Fairness  Restoration  Good relations | **Goal** |  |
|  | Loss of peace  Animosities  Know each other | **Consequences** |  |
|  | Forgiveness  Confrontation  Common ground | **Management process** |  |
